# Osteopathic Manipulative Treatment for Chronic Low Back Pain and Musculoskeletal Pain in Remote Underserved Populations: A Systematic Literature Review

**DOI:** 10.7759/cureus.83524

**Published:** 2025-05-05

**Authors:** Arielle Friedman, Reena Sheth, Jill Wallace-Ross

**Affiliations:** 1 Osteopathic Medicine, Nova Southeastern University Dr. Kiran C. Patel College of Osteopathic Medicine, Fort Lauderdale, USA; 2 Foundational Sciences, Nova Southeastern University Dr. Kiran C. Patel College of Osteopathic Medicine, Fort Lauderdale, USA; 3 Family Medicine, Nova Southeastern University, Fort Lauderdale, USA

**Keywords:** low back pain, medically-underserved areas, musculoskeletal pain, osteopathic manipulative medicine (omm), osteopathic manipulative treatment (omt), outreach medical camps, remote populations, rural and underserved communities, telehealth

## Abstract

Chronic musculoskeletal pain, particularly chronic low back pain (CLBP), is a prevalent issue among rural populations, where limited access to healthcare exacerbates the condition and significantly impacts various aspects of daily life. The effects of chronic pain and healthcare barriers extend beyond physical health, influencing mental well-being, physical activity, and overall functionality as rural populations age. This complex healthcare challenge requires further investigation to identify effective, culturally relevant, and cost-efficient solutions. Proposed approaches include osteopathic manipulative treatment (OMT), telehealth, and alternative education models designed to incentivize healthcare professionals to serve these communities while respecting their cultural values and societal needs. Medical missions and volunteer healthcare efforts are already making strides in alleviating the impact of CLBP, which can lead to mental health decline, reduced mobility, and functional impairments. This systematic literature review synthesizes recent research on the effectiveness of OMT in managing chronic musculoskeletal pain, with a particular focus on low back pain (LBP) in underserved and rural populations. It examines how biopsychosocial factors and healthcare access influence treatment outcomes, while also proposing strategies for improving healthcare access through innovative education models and the use of technology. The review also considers the importance of community engagement and cultural sensitivity in these approaches. By analyzing research articles published between 2013 and 2023 from Google Scholar and PubMed, along with additional articles identified through a manual search of the *Cureus* journal website, this review evaluates 26 relevant studies on the therapeutic benefits of OMT for CLBP. It aims to highlight the potential of OMT as an effective intervention for rural populations and to better understand the broader implications of chronic musculoskeletal pain on these communities.

## Introduction and background

Chronic musculoskeletal pain, especially low back pain (LBP), is a major public health issue which affects millions of people worldwide and leads to significant disability, diminished quality of life (QoL), and increased healthcare costs [1]. Managing this condition is especially challenging in rural and underserved communities, where healthcare resources including access to specialists and specialized treatments are limited [2]. Current clinical management guidelines for chronic low back pain (CLBP), defined as over three months of persistent LBP, emphasize non-pharmacologic approaches such as physical therapy to improve mobility and functionality, cognitive behavioral therapy (CBT) to manage pain-related distress, and patient education on lifestyle modifications; however, these treatments require frequent in-person care which may not be feasible in remote areas [1]. Rural populations also face many intersectional challenges, such as poverty, lower levels of education, and higher rates of comorbid conditions, which further complicate pain management [2]. These compounding issues highlight the need for alternative, effective and accessible treatment options that address not just the physical, but also the social and psychological aspects of chronic pain. Osteopathic Manipulative Treatment (OMT), a hands-on therapy provided by Doctors of Osteopathic Medicine (DOs) and by Doctors of Allopathic Medicine (MDs) with specialty training, represents a potential non-invasive, non-pharmacologic solution that aligns with the holistic, patient-centered care needed for proper pain management in underserved populations [3].

OMT involves applying manual pressure or force to the body to restore functionality, reduce pain, and promote healing [4]. The technique is grounded in osteopathic principles that emphasize a connection between the body’s structure and its function [4]. Research has demonstrated OMT’s effectiveness in managing both acute LBP and CLBP by improving pain, functional status, and overall QoL [5-8]. These beneficial outcomes could be advantageous for populations where access to conventional treatments is limited, such as those living in rural or low-income areas. The American Osteopathic Association (AOA) recommends OMT for patients with both acute LBP and CLBP, supporting its effectiveness based on high-quality evidence from clinical trials [9].

Osteopathic medicine’s whole-person approach to patient care can be an effective treatment strategy for addressing the multifaceted needs of rural and underserved populations. DOs are trained to understand the interconnectedness of the body’s systems and to use their hands to both diagnose and treat pain, which makes them especially well-equipped to manage musculoskeletal conditions like LBP [4,10]. The hands-on, interpersonal nature of OMT not only targets the physical aspects of pain but also addresses underlying biopsychosocial factors such as anxiety and depression that are known to worsen the experience of pain, making it an even more valuable tool for patients in underserved areas [8,11].

Despite growing evidence supporting OMT’s effectiveness, it remains an under-recognized and under-utilized treatment modality. Barriers to widespread adoption include limited recognition of OMT as a standard treatment, a restricted scope of practice for DOs in certain countries, and a lack of basic education and training on OMT among healthcare professionals [4,12]. Additionally, while OMT holds significant promise, it is not the only solution for improving access to musculoskeletal pain management in rural and underserved areas. To overcome logistical challenges in reaching these communities, telemedicine has emerged as a complementary tool that can enhance access to follow-up care after OMT treatments, education, and remote monitoring, particularly for populations with limited access to in-person healthcare services. Furthermore, education-based models that incentivize healthcare professionals to serve in remote regions can also play a crucial role in addressing disparities.

The aim of this review is to investigate the effectiveness of OMT in managing chronic musculoskeletal pain, particularly LBP, in underserved and rural populations. This review not only focuses on the potential of OMT for chronic musculoskeletal pain management in rural populations but also considers the broader landscape of innovative solutions, including telemedicine, to improve healthcare access and outcomes in these communities. Additionally, this paper will explore how OMT can be incorporated into healthcare systems serving these populations and how it can help bridge the treatment gap for those facing barriers to conventional care, with special consideration given to factors like healthcare access, socioeconomic status, and community support. With its holistic, hands-on approach, OMT represents a promising, sustainable alternative for chronic pain management, offering the potential to improve both physical and emotional well-being in underserved regions around the world.

## Review

Methodology 

**Figure 1:**
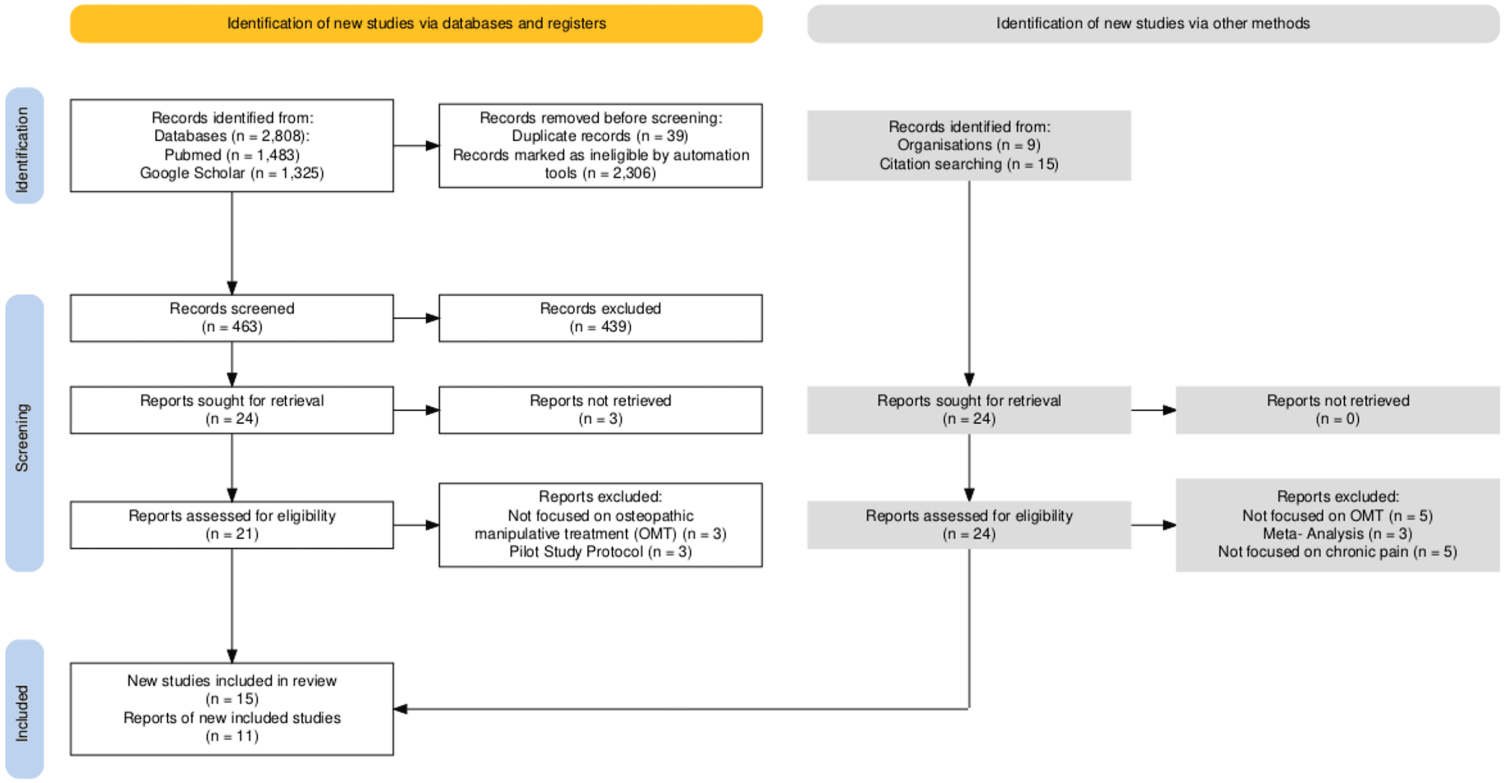
Study selection Preferred Reporting Items for Systematic Reviews and Meta-Analyses (PRISMA) flow diagram [13]

This systematic literature review outlines current evidence regarding the effectiveness of OMT for the management of chronic musculoskeletal pain with an emphasis on LBP across different populations, including those in remote, underserved regions, as well as biopsychosocial factors influencing treatment outcomes and accessibility. The databases used were Google Scholar and PubMed. In addition, we manually searched the *Cureus* journal website to identify any relevant articles not captured through database searches. The review was conducted under Preferred Reporting Items for Systematic Reviews and Meta-Analyses (PRISMA) Guidelines. The search terms: “Latin (South) America,” “Healthcare Access,” “Low Back Pain,” “Chronic Pain,” “Osteopathic Manipulative Treatment,” “Osteopathic Manipulation,” “Musculoskeletal Pain Disorders,” “Quality of Life,” “Remote Access to Healthcare,” and “Rural Communities” were used to find relevant articles. Inclusion criteria permitted original studies conducted on human participants in any part of the world published between 2013 and 2023, cross-sectional studies with a sample size greater than 20, experimental studies, longitudinal studies, narrative reviews, and descriptive studies, as well as studies reporting on the mechanisms of LBP, chronic musculoskeletal pain in rural communities, and the usefulness of OMT to mitigate the impact of musculoskeletal pain. Although language was not an explicit inclusion criterion, all studies ultimately included in this review were published in English. The aforementioned researchers carried out all searches. Studies were excluded if they were classified as pilot study protocols, meta-analyses, or scoping reviews. Studies published in non-peer-reviewed books or documents were also excluded. The systematic review of pertinent studies and the extraction of important data, such as study design, sample size, participant demographics, intervention details, outcome measures, and findings, were followed under PRISMA criteria. Data was synthesized to identify patterns and themes across eligible studies. Limitations to this methodology include the broad scope of findings and variety of studies, as well as the exclusion of meta-analyses and scoping reviews. Additionally, only studies published in English were included, which may introduce language bias and limit the inclusion of relevant research from non-English-speaking regions. The PRISMA flow diagram in Figure 1 illustrates the study selection process used in this systematic review.

Discussion

Pain Prevalence

Overall, findings from five studies conducted in remote areas of Vietnam, Peru, Chile, Brazil, and the Amazon riverine suggest that chronic musculoskeletal pain is prevalent in rural regions across Vietnam and South America. More specifically, a high prevalence of chronic back pain exists in various rural populations across South America. Additionally, chronic back pain is particularly common among middle-aged and elderly women.

A secondary analysis of a 2009-2010 Chilean National Survey found that 42.6% of Chilean participants self-reported chronic musculoskeletal symptoms. Overall, the prevalence of chronic musculoskeletal symptoms in Chile was greater than the national prevalence of dyslipidemia, hypertension, respiratory symptoms, depression, and type 2 diabetes [14]. Increased chronic disease prevalence across many remote regions is coupled with increased pain burden: a 2015 survey-based descriptive study in remote Peruvian Amazonian communities revealed more frequent reports of daily musculoskeletal pain secondary to heavy lifting, most commonly cited by women compared to men. Nearly all participants (98%) also reported non-traumatic, non-accident-related musculoskeletal pain, and over half of the participants reported experiencing pain daily [15]. Additionally, anonymous questionnaires completed by patients of medical outreach programs in Vietnam and South America revealed a high overall prevalence of musculoskeletal pain among women and elderly populations. Acute LBP and chronic upper back pain were most frequently self-reported by South American participants compared to Vietnamese participants, particularly by females with an average age of 44. Of this group, 29% were homemakers, and 11.8% were agricultural workers [16]. Results from a self-reported survey in Brazil revealed that LBP affects approximately one-fifth of the Brazilian population, with higher reports among women. Furthermore, the prevalence of chronic pain increased gradually and proportionally with age. The authors hypothesized that higher reports of LBP among women may be due, in part, to greater intensity of housework, increased exposure to repetitive tasks, non-ergonomic positions, working at high speeds, and pregnancy/postpartum status [17]. Lastly, a cross-sectional survey of elderly populations living along the rural Amazon riverine found that 50% of men and women aged 60 and older reported chronic back pain, with the average onset of pain starting at age 43. Among participants with chronic back pain, most had not sought or received any related treatment [18].

The findings summarized above represent the current state of literature on the prevalence of chronic musculoskeletal pain and chronic back pain among remote populations. Evidently, chronic musculoskeletal pain is a significant burden in rural communities across Vietnam and South America, particularly chronic back pain across South America. However, details pertaining to the character and anatomic location of chronic back pain in these populations remain understudied. There seems to be a clear scarcity of epidemiologic data defining the extent of musculoskeletal pain and overall health status in many rural regions worldwide, especially when compared to the vast body of existing literature on chronic back pain in general. The lack of available data represents a substantial gap in what is otherwise a well-studied topic. Future studies should be conducted across a wider geographic range of remote populations to realistically illustrate the true prevalence, nature, and anatomic location of chronic pain. If possible, data should be collected and categorized on a country-by-country basis to more accurately discern regional differences in pain prevalence rather than generalizing results to an entire continent. Future studies should also utilize validated measures of disability such as the Roland-Morris Disability Questionnaire (RMDQ) or the Oswestry Disability Index (ODI), to objectively capture participants’ subjective pain experiences. Obtaining a more detailed understanding of the problem will help pave the way for more accurate diagnosis and management of somatic dysfunction and chronic pain in rural, underserved populations.

Pain Risk Factors

Several studies demonstrate biological, psychological, geographical, and socioeconomic associations with back pain prevalence, severity, and disability. The most common overlapping findings include advanced age, female gender, multiple pain sites, a history of pain, health comorbidities, poor sleep, heavy physical activity, insufficient physical activity, low education, and low income.

Anonymous questionnaires completed by patients of medical outreach programs in Vietnam and South America reveal that older age is associated with an increased number of musculoskeletal complaints and a lower self-rated health status. Conversely, a lower self-rated health status is associated with increased reports of both acute and chronic pain [16]. Results from a self-reported survey in Brazil reveal that, in both men and women, chronic back pain is significantly associated with a low education level, increased length of heavy physical activity at work and home, history of smoking, high salt intake, being overweight or obese, and having hypertension and/or high cholesterol. Specifically, for Brazilian men, being 65 years old or older and living in a rural area are significantly associated with chronic back pain. For Brazilian women, being between the ages of 55 and 64 and regular candy consumption are significantly associated with chronic back pain. Additionally, consuming the recommended daily number of fruits and vegetables protects women from back pain [17]. Secondary analysis of a Brazilian National Health Survey from 2019 identified biological factors associated with increased LBP, including being female, overweight or obese, a current smoker, elderly, or having other chronic conditions; psychological factors such as depression; and social factors such as low education level, low household income, and living in rural areas. Additionally, individuals with low education, low income, obesity, depressive symptoms, current cigarette use, and/or other chronic conditions are more likely to have limited activities of daily living secondary to CLBP [19]. A 2017 study comparing characteristics between Brazilian and Dutch adults aged 55 and older who presented with a new episode of back pain highlights important sociocultural and lifestyle factors associated with increased pain severity and disability: irrespective of country, increased pain severity is associated with female gender, poor sleep quality, low education level, having two or more comorbidities, and having a greater number of pain sites [20]. Increased back pain-related disability is associated with poor sleep quality, low education level, prior history of pain, having two or more comorbidities, having a higher body mass index, and having a greater number of pain sites, also irrespective of country [20]. The study also uncovers several country-specific differences: Brazilian adults exhibit greater symptom severity and disability, as well as increased depression and pain catastrophizing, compared to Dutch adults [20]. This particular finding suggests that country of residence is an important determinant of disability level and may also influence the physical and psychological burden of back pain in older adults.

Knowledge of factors generally associated with back pain and those associated with higher degrees of pain severity and disability can help guide the management of back pain in a context-dependent manner and decrease overall prevalence. Importantly, many of these factors are modifiable and should be taken into account by healthcare providers when considering management strategies for older adults. For example, public policies that aim to reduce obesity should be prioritized because weight loss can decrease pain and disability [17]. Knowledge of these factors can also aid in the development of context-dependent prevention strategies in high-risk populations, potentially leading to a decrease in incidence. A limitation of the studies discussed above is that they are cross-sectional and thus lack the longitudinal data necessary to demonstrate causality. For causal associations to be made, future studies should retrospectively assess the impact of prior factors on current back pain incidence, severity, and disability, or prospectively assess the impact of current factors on future back pain incidence, severity, and disability.

Treatment Barriers

A review of nine studies demonstrates an overarching prevalence of barriers to the effective treatment of CLBP and musculoskeletal pain across rural and remote, underserved regions of Australia, Brazil, Ecuador, India, Nepal, and the Peruvian Amazon. Cultural barriers include language differences, gender inequalities, and a misperception of the emphasis on traditional medicine - such as herbal treatments, spiritual or ritual practices, and other culturally rooted healing methods - over modern, biomedical approaches like pharmaceuticals and surgical interventions in some communities. Geographic barriers include community remoteness, distantly located health camps with limited supplies, and the maldistribution of healthcare workers in urban-centered programs. Socioeconomic barriers include increased poverty and decreased education. System-level barriers, such as flawed universal healthcare initiatives, urban-centered medical schools, scarcity of remote-based research, and understaffed health camps, also play a considerable role.

Cultural barriers to treatment: Outcomes from a 2015 survey-based descriptive study assessing health, health knowledge and practices, and healthcare access in remote Peruvian Amazon communities receiving care from a mobile medical clinic shed light on the health-related consequences of gender inequalities. In these regions, gender inequality between men and women is a sociocultural norm that contributes to poor health-seeking behavior. Compared with men, women have lower education levels, are less likely to be single, and are more likely to be married [15]. The combination of these factors decreases financial independence among female community members, ultimately contributing to poorer health-seeking behavior [15]. This study also reveals that community members are more likely to utilize modern medicine over traditional medicine: the majority (72%) of individuals reporting pain are actively managing it with modern medical therapy [15]. Furthermore, most participants (75%) say they would rather use modern analgesics over traditional therapies to relieve their pain; however, if prescribed traditional or modern medicine, a little less than half (48%) say they would be equally compliant with both [15]. Participants are also more likely to have ever refused traditional medicine. Most (85%) of individuals who had ever declined traditional medicine cite mistrust and/or disbelief in traditional medicine as their primary reason for refusal [15]. In contrast, a 2023 editorial outlining the challenges of delivering healthcare to remote tribal communities in India describes a cultural milieu that emphasizes traditional healing and distrusts Western medicine [21]. The variation in existing attitudes toward traditional versus modern medicine suggests that cultural ideologies surrounding healthcare are evolving in remote areas. Because of this evolving cultural landscape, the assumption that remote communities do not want modern medicine hinders the development of targeted healthcare initiatives and future health policy and planning in underserved regions. Of note, remote communities closer to nearby cities may have increased exposure to modern medical practices, which influences their preference for modern over traditional medicine-while those living in extremely remote areas may still display a preference for traditional practices [15]. Evidently, more research is needed to better understand varying beliefs across indigenous communities in order to potentially incorporate elements of traditional medicine into local healthcare provision alongside modern health promotion and education [15]. Given its hands-on, non-pharmacologic approach, OMT may hold cultural resonance in communities that value traditional healing practices, presenting an opportunity for greater cultural acceptance when integrated thoughtfully into care models.

Language barriers also pose significant challenges to healthcare delivery, specifically in tribal areas of India. For example, limited access to health information in native languages prevents tribal community members from making informed health decisions [21]. Language differences also obstruct effective communication between health providers and tribal patients, leading to misunderstandings and suboptimal outcomes [21]. Providing language interpretation services in these communities can improve communication and understanding, ultimately leading to more culturally competent care, better health outcomes, and reduced health disparities [21].

Geographic barriers to treatment: Geographical isolation represents a significant barrier to healthcare in remote communities across Nepal, India, Brazil, and the Peruvian Amazon [15,19,21-23]. In communities in the Peruvian Amazon, healthcare access and healthcare-seeking behavior are limited by long distances (often over two hours) to the nearest health post, as well as long waiting times upon arrival [15]. Distant health camps also present challenges for consistent follow-up opportunities between patients and providers [15,22]. Limited infrastructure and geographical isolation further complicate the timely delivery of medical supplies, impeding access to certain treatments [21]. For example, health camps in remote Nepal can only provide a narrow range of prescription medications due to formulary constraints [22]. The limitations in available treatment options ultimately increase the risk of poorly controlled pain and disease. A 2023 cross-sectional study of two Ecuadorian cohorts found that older women are more predisposed to require surgery for lumbar disc herniation, suggesting that older individuals with certain health conditions may need more intensive treatment regimens compared to younger individuals [24]. Advanced treatment options, especially surgical interventions, are particularly difficult to obtain in geographically remote, underserved areas of the world. Furthermore, two studies report a maldistribution of healthcare services across Peru and India, with most general and specialized services concentrated in urban areas [15,23]. In Brazil, secondary analysis of a national health survey in 2019 revealed regional differences in treatment modalities for CLBP: residents living in urban areas are more likely to report using all types of treatments, except for alternative methods [19]. Current healthcare initiatives in Peru are also largely focused on accessible, urbanized regions [15]. These initiatives require redesign to be implemented in rural settings, but such redesign is hindered by chronic underfunding, poor staffing, and a lack of training [15].

Socioeconomic barriers to treatment: Outcomes from six publications suggest a relationship between low socioeconomic status, low education, and a high prevalence of chronic pain across many remote populations worldwide. In remote regions of Nepal, 25% of the population lives below the poverty line [25]. Poverty is also widespread in the remote Peruvian Amazon, with 42% of community respondents living on less than $1.25 U.S. dollars per day, 35% going to bed hungry in the past month, and 23% reporting household debt [15]. Consequently, impoverished individuals struggle to meet their basic needs and often neglect medical care [21]. With limited financial resources for travel, the first healthcare service sought is typically whatever is available in the community [18]. The high cost of in-person medical care, coupled with general medical care neglect, worsens the burden of chronic disease, leading to even greater financial strain, such as the need for frequent follow-up appointments and more intensive treatments. The poverty-to-poor-health cycle is evident in a 2021 retrospective analysis of data collected from a medical outreach trip to remote Nepal, where 21% of patients seen had unmet health needs, 11% required specialty services beyond the capability of the health camp, and 6.8% required regular follow-up managed by a permanent clinic [22]. In addition to increased financial burdens, remote communities often receive limited education. Limited education reduces community awareness of preventative healthcare measures, ultimately increasing susceptibility to preventable illnesses [21]. It also leads to lower health literacy and a general lack of knowledge about how to effectively utilize available healthcare systems. Surveys conducted in a remote Peruvian Amazon community reveal that 85% of individuals who have ever been formally diagnosed with an acute or chronic disease do not fully understand their diagnosis, though they are eager to learn more [15]. In Brazil, a secondary analysis of a national health survey in 2019 shows that urban residents tend to be more educated, which may explain their higher use of healthcare services [19]. Those with better socioeconomic conditions are also more likely to receive treatment, despite Brazil offering universal health care, highlighting the need for tailored policies and prevention programs that pay special attention to vulnerable groups [19].

System-level barriers to treatment:System-level barriers to treatment broadly consist of flawed universal healthcare systems, a scarcity of remote-based research, urban-centered medical schools, and a severe shortage of qualified medical professionals in remote regions. The Peruvian government developed a universal healthcare system over a decade ago to reduce the nation’s health disparities; however, marginalized and isolated communities in Peru are unable to access this system due to a lack of knowledge and/or proper documentation, such as a national identity card [15]. As a result of Peru’s healthcare system failing to recognize the unique needs and challenges of certain populations, current non-governmental healthcare provision in the remote Loretto region mainly consists of mobile medical boat clinics, traditional medicine, healers called *curanderos*, or a combination of modern and traditional practices [15]. Furthermore, the redesign and reimplementation of healthcare initiatives in rural settings is hampered by chronic underfunding, poor staffing, and a lack of clinician training [15]. In addition to poorly planned government initiatives, there is a scarcity of research in Peru regarding health, health beliefs, and healthcare access in remote communities [15]. Because of this limited remote-based research, there is a poor understanding of population-specific healthcare needs, ultimately leading to fewer treatment initiatives.

In rural and remote areas of Nepal, disparities in healthcare access and service quality stem from ineffective medical education, inefficient deployment, and poor retention of health human resources. As of 2016, health professions education in Nepal is predominantly urban-centered and encompasses curricula that fail to address medical education within the context of rural communities [25]. There are also unmet needs among health workers and medical students for real-life experience working in rural areas of Nepal, as well as a need for positive attitudes from those who do work in rural areas [25]. Urban medical schools' failure to recognize the unique needs, challenges, and values of healthcare practice in remote Nepalese communities leads to a lack of advocacy for these communities on both the micro (local) level and macro (policy) level [25]. Nepal also has significant urban-rural disparities in terms of access to health services and the quality and impact of available services: two-thirds of Nepal’s physicians are concentrated in urban areas, while rural healthcare facilities remain chronically understaffed [25]. A retrospective analysis of data from patients who attended a health camp in rural Nepal in 2017 reveals similar findings: Nepal has 0.598 physicians per 1000 people, but only 0.019 physicians per 1000 people in rural areas [22]. When compared to the World Health Organization’s recommendation of one physician per 1000 people, the significant shortage of qualified medical professionals in remote areas becomes evident [22]. Similarly, in India, a 2023 literature review states that a persistent barrier to primary care is the shortage of qualified medical professionals in the country [23]. Lastly, a 2019 qualitative analysis of interviews with Australian and international medical graduates, medical supervisors, and practice managers found that Australians living in rural and remote areas have access to 274 doctors for every 100,000 people, while Australians in major cities have access to 433 doctors for every 100,000 people [26]. Moreover, there is no consistent framework for medical supervision across the various remote Australian communities [26]. These results suggest that the maldistribution of health workers continues to be problematic and undermines the capacity to achieve health improvements for rural and remote populations.

Pain Impact

LBP is a common issue affecting individuals across various age groups, significantly impacting QoL through both physical and psychological factors. Four articles demonstrate this concept, suggesting a need for further investigation into ways to mitigate this community-wide issue. The impact that chronic pain has on a population can be divided into three categories: the negative impact on physical and mental QoL, the impact of pain with increased age, and the loss of productivity due to pain.

Impact on QoL:A cross-sectional study, based on a secondary analysis of a musculoskeletal disease survey in Chile, demonstrates that the presence of chronic musculoskeletal symptoms is a risk factor for low health-related QoL across both physical and mental dimensions. These include physical function, ability to carry out activities of daily living, body pain, general health, vitality, social functioning, emotional functioning, and mental health. Low scores are found in both the mental and physical dimensions when it comes to chronic musculoskeletal symptoms in the Chilean population. This is consistent with research assessing health-related QoL through the survey, which suggests that QoL is reduced in chronic pain sufferers, even when the symptom intensity is low. Furthermore, the paper states that 62.8% of the rural population presents with a “low health-related QoL” in the physical health dimension, indicating that the impact is greater in rural populations [14]. Another study shows that among those with chronic back pain, 20% report having depressive symptoms [19].

Impact on older populations: An observational study confirms that chronic musculoskeletal pain lowers QoL and adds that elderly individuals with musculoskeletal pain, particularly those with more pain sites, have lower physical performance. Additionally, pain in older adults is associated with adverse beliefs about back pain and poor mental health [20]. A cross-sectional study reveals that lower self-reported health scores and higher numbers of musculoskeletal complaints are associated with older age, highlighting the prominent effect of somatic dysfunction on QoL in the elderly population. Furthermore, underdeveloped nations bear the greatest burden of musculoskeletal disease worldwide, which likely contributes to the overall poor health status often encountered in settings of poverty [16]. Since many rural communities are composed of older populations, with younger residents seeking opportunities elsewhere, it is important to understand that older members of these communities are more severely impacted by chronic musculoskeletal symptoms.

Impact on productivity: Acute and chronic back pain are the most prevalent types of pain reported by landscapers and farmers living in rural populations across South America and Vietnam. Most notably, there is a high incidence of musculoskeletal symptoms within the sampled populations: all respondents report either acute or chronic pain in at least one body part [16]. This finding suggests a correlation between musculoskeletal pain, rural lifestyles, and occupation. A cost analysis study in Chile emphasizes that LBP is associated with highest costs of all musculoskeletal diseases due to economic losses for governmental institutions from productivity declines and increased sick leave. Specifically, LBP has the highest cost among all other diseases ($187,908,323), representing 27.4% of the overall cost associated with musculoskeletal pain. The high number of sick leaves taken (273,297─with a total of 2,324,466 paid sick leave days) reflects a loss of productivity due to LBP and the subsequent monetary burden [27]. Furthermore, this study suggests that LBP is a multifaceted health problem due to its psychological impact on patients, system-level barriers to diagnosis and management, and social shortcomings [27].

Understanding the impact of pain on elderly populations, mental and physical QoL, and productivity aids in the formation of individualized, multifaceted treatment plans, as opposed to focusing solely on symptomatic management, leading to better overall outcomes. This holistic view lends itself to the use of OMT, which focuses on treating the mind, body, and spirit as a whole.

OMT

Outcomes from nine studies demonstrate OMT’s efficacy in treating CLBP, highlighting its potential as a clinically effective, cost-effective, and sustainable treatment option for patients suffering from CLBP. The use of OMT for chronic musculoskeletal disorders, particularly CLBP, is a well-studied topic; however, its practical application in rural populations has yet to be fully explored. Within the context of underserved rural populations, OMT can serve as an innovative method for treating and preventing musculoskeletal disease, as well as an alternative to conventional medical therapies, which often focus solely on the symptomatic treatment of disease. Due to its simplistic, hands-on nature, OMT is also considered an ideal care option for resource-scarce areas.

Evidence of OMT efficacy in CLBP:CLBP is prevalent among the working-age population and imposes a significant burden by hindering daily activities and contributing to mental distress as demonstrated above, thus this makes it a multifaceted issue that OMT as a treatment can lend its strengths to. Results from a randomized trial reveal significant reductions in nonspecific CLBP and functional disability in patients receiving OMT, specifically when using techniques targeting the diaphragm. The study also reports a statistically significant reduction in pain catastrophizing in the OMT intervention group with diaphragmatic techniques compared to the sham manipulation group which only used OMT, as assessed using the Spanish Pain Catastrophizing Scale (PCS). Additionally, the OMT intervention group had a statistically significant reduction in anxiety and depression at both four and 12 weeks compared to the sham group, as assessed using the Hospital Anxiety and Depression Scale (HADS) [28].

Primary outcomes from another randomized double-blind study further emphasize OMT’s effect on pain reduction: subjects experienced substantial LBP improvement, with over 50% pain reduction measured via the RMDQ. The study also concludes that OMT is most effective for patients with severe CLBP. Overall, OMT appears to be an efficacious, low-cost, minimally invasive initial treatment option for patients with severe CLBP and should be considered before proceeding to more invasive and costly treatments [29]. Rotter et al. reinforce OMT’s usefulness for managing CLBP, with subjects showing improvements in pain intensity and function for at least 12 weeks. The study notes a comparison of clinical significance with a minimally clinically important difference (MCID) of 1.2 points improvement in overall patient satisfaction with treatment that would warrant a change in their treatment choice in the future between OMT and other treatment options. This MCID score further highlights OMT as efficacious but not necessarily more efficacious than conventional treatment [30].

Recent studies in 2023 continue to emphasize OMT’s effectiveness for managing CLBP, reinforcing earlier findings that OMT can provide sustainable pain management and is a better option compared to other manipulation techniques. Lizis et al., in a randomized control trial comparing OMT to Kaltenborn-Evjenth Orthopedic Manual Therapy (KEOMT), demonstrates statistically significant improvements in pain severity, disability, and overall health status in subjects receiving OMT compared to those receiving KEOMT. This suggests that OMT is highly efficacious for CLBP. Notably, 82.3% of participants had minimal disability after OMT treatment compared to only 20.6% of participants in the KEOMT group, as assessed using the Numerical Pain Rating Scale (NPRS) [31]. Another recent study reveals that OMT users report significantly better results regarding pain intensity, pain impact, physical function, and health-related QoL for 12 months compared to non-users, and that OMT integrated into conventional medical care has the most long-term benefit [32]. Thus, OMT integrated within standard medical care provided by DOs for the improvement of CLBP should be utilized by the growing osteopathic physician workforce [32].

Mechanisms behind OMT effectiveness:OMT's underlying neurophysiological, psychosocial, and emotional mechanisms help mitigate the negative effects of chronic musculoskeletal diseases, specifically CLBP. Salvador et al. and Lizis et al. discuss the neurophysiological mechanisms behind OMT, explaining that manual manipulation enhances muscle tension and joint mobility while inhibiting neural function of afferent pain pathways, leading to pain relief [28,31].

Salvador et al. also establish an association between OMT and reduced fear avoidance behaviors, as well as between OMT and reduced pain catastrophizing. Fear avoidance-the belief that avoiding certain activities will reduce pain-was significantly reduced in the diaphragm OMT group at both four and 12 weeks when compared with the sham intervention group which also received OMT, but without any diaphragmatic intervention [28]. By minimizing fear avoidance and pain catastrophizing, which is an exaggerated cognitive response before, during, or after a painful event associated with ruminations, magnification, and helplessness, individuals with CLBP can reduce pain, leading to improvements in physical, mental, and overall lifestyle [28]. Conventional treatments such as medication, surgery, or physical therapy lack the multifaceted benefits seen in patients receiving OMT, making it a promising holistic treatment option, as demonstrated in the studies above.

Long-term efficacy and sustainability: In a 2014 study of 54 patients who showed an initial clinical response to OMT prior to week 12, the majority (76%) remained symptom-free at their week 12 exit interview, demonstrating OMT's usefulness in the management of LBP for up to 12 weeks [33]. The same study concludes that OMT may also help prevent symptom relapse in non-depressed patients experiencing longer durations of LBP [33]. A recent retrospective cohort study shows that OMT's effects on CLBP can last as long as 12 months, with sustained positive effects on pain, disability, and QoL [32]. The study suggests that integrating OMT with conventional treatments for CLBP is linked to positive outcomes; with the growing workforce of DOs in the U.S., its use could be more widely facilitated when aligned with clinical practice guidelines [32]. Overall, OMT is proven to be a sustainable and potentially long-term solution for CLBP [33].

Cost effectiveness:CLBP is often managed with costly or invasive interventions, including opioid analgesics, epidural corticosteroid injections, and spinal surgery, particularly in patients with higher pain severity. OMT has been shown to be a more cost-effective adjunctive treatment for improving physical function and QoL in patients with LBP and joint pain when compared to other specialties such as orthopedics, neurology, physical therapy, or acupuncture [34]. OMT’s unique patient-centered focus, which uses health-oriented principles of patient care and skills-including hands-on manual diagnosis and treatment-may help reduce the burden of musculoskeletal conditions and healthcare costs.

When compared to usual care, OMT performed by trained osteopaths has also been found to be a dominant and cost-effective strategy for managing LBP. In a cost-utility analysis for LBP, osteopathy resulted in cost savings and improved quality-adjusted life years compared to usual care [35]. Overall, OMT is an effective and cost-friendly method for treating CLBP due to its minimal tool use, decreased reliance on medication, and reduced need for advanced equipment or imaging. Because of its cost-effectiveness, OMT is an excellent treatment modality for rural communities that cannot afford advanced medical technology or abundant medical supplies.

In summary, nine peer-reviewed articles detail the effectiveness, sustainability, mechanisms, and cost benefits of OMT. Osteopathic medicine’s holistic approach can be used effectively to manage chronic musculoskeletal diseases while also reducing secondary effects. Recent evidence supporting the use of OMT suggests that this method of treatment could be applied to broader populations, specifically those in rural or healthcare-inaccessible areas.

Future Considerations

Rural healthcare challenges include distance, limited provider access, and healthcare disparities. Overcoming these challenges requires collaborative efforts between the government, medical specialists, and the rural communities themselves. A review of seven studies suggests that telehealth, when appropriately implemented, can address healthcare access in rural and remote areas. However, successful outcomes require overcoming technical and social barriers, alongside innovative community-based education and outreach efforts.

The role of telehealth in reducing rural healthcare barriers:One of the main benefits of telehealth is improving access to healthcare providers. In a descriptive study in rural Italy, a teleassistance service for frail elderly individuals showed that telehealth improves accessibility, though user satisfaction depends on usability for older populations. The teleassistance service was positively received by the majority (85%) of interviewed participants, with 75.6% stating they would recommend the service to friends and acquaintances [36]. A randomized controlled trial providing telerehabilitation for CLBP patients in Nigeria shows that telehealth interventions are not only cost-effective but also reduce barriers to accessing physiotherapy in remote areas [37]. According to a narrative review, telemedicine has the potential to expand healthcare access in remote areas of India by bypassing geographical barriers and reducing travel time for patients. The review specifically states that telemedicine’s benefits include flexible work schedules, high patient satisfaction, increased access to high-quality specialist care, and cost savings for both patients and healthcare systems [23].

Telehealth is more affordable compared to in-person healthcare, particularly in developing countries, due to significant savings in travel and clinic costs. A randomized controlled trial found a statistically significant cost difference between clinic-based interventions and telemedicine-based services, with clinic-based participants paying an average of $44.30 more per person over the eight-week treatment period. Additionally, telemedicine-based interventions cost approximately 50% less than clinic-based interventions due to fewer clinic-based requirements and less frequent use of physiotherapists for treatment delivery [37]. Furthermore, telemedicine has the potential to lower healthcare costs by eliminating the need for physical infrastructure in rural areas [23].

The role of community engagement and education in rural healthcare:According to outcomes from a short-term medical service trip, the most common adult complaints in remote Amazonian communities are neck and back pain secondary to physically demanding tasks such as harvesting and lifting, as well as skin and soft tissue infections secondary to trauma or water exposure [15]. The healthcare outcomes for such complaints seen in rural communities can be improved by simulation-based education methods, community-based learning education models, and by integrating traditional medicine with modern healthcare.

The use of simulation-based learning to train healthcare professionals in rural and remote settings can improve the quality of healthcare in underserved areas. Outcomes from a 2022 study reveal that learning management systems are well-received by workshop attendees, who find them to be a useful tool. Additionally, attendees appreciate the ability to watch videos prior to practicing on simulators [38].

The Patan Academy of Health Sciences (PAHS) is a rural-based education model developed in Nepal that promotes rural health by embedding medical education in rural community settings. It encourages learning within a rural context, fosters opportunities for community and peripheral health workers to participate in teaching, learning, and evaluating medical students, and involves community members in curriculum design and implementation. The PAHS model can improve rural health by training medical students through real-life experiences in rural areas, helping them develop a positive attitude and affinity for working in rural communities after graduation. A key marker of PAHS’s success in advocating for rural primary healthcare is the willingness and dedication of PAHS graduates to serve the communities they pledge to work in. So far, PAHS has engaged with communities with numerous successes and no major associated problems [25]. Other proposed solutions to enhance tribal healthcare delivery in India include increasing community participation to strengthen the cultural relevance and effectiveness of healthcare education programs, expanding mobile clinics and outreach trips in rural areas, promoting health education and awareness, and practicing culturally-sensitive medicine [21].

Healthcare outreach should engage local communities and integrate both systems for more inclusive care. A recent study in remote Amazonian communities suggests that local healthcare posts can boost health promotion by engaging locals to uncover and dispel health misconceptions, as well as offering simple educational interventions like leaflets and pamphlets. Increasing indigenous healthcare worker training is also crucial [15]. Roy et al. build on this idea, proposing that in tribal areas, culturally-sensitive healthcare models that integrate traditional practices with modern medicine can lead to better health outcomes [21]. Additionally, health education should focus on preventive healthcare measures, disease management, and hygiene practices tailored to the cultural and linguistic context of the community [21]. Overall, encouraging community-based education and engagement in rural areas can strengthen the relationship between healthcare providers and local communities.

Challenges in telehealth:As telemedicine usage continues to grow, healthcare providers must remain mindful of the new challenges it brings. A 2020 descriptive study assessing telehealth usability and adherence among older populations highlights the importance of user-friendly design in telehealth systems, with particular attention paid to elderly groups [36]. The results from this study reveal a high incidence of technical challenges and usability problems among elderly participants, with many struggling to complete telehealth services. Furthermore, 70% of participants encountered technical problems that could easily be resolved with technical support. Another challenge is that telehealth's success and patient engagement depend on internet connectivity, which can be an issue for remote communities lacking existing infrastructure [37]. Limited access to technology, coupled with reduced technological literacy, can further exacerbate healthcare inequities in many rural and remote communities [23].

Overall, while telehealth offers significant potential for addressing rural healthcare challenges by improving access, reducing costs, and overcoming geographic barriers, its effectiveness depends on addressing technical challenges and integrating community engagement. Ongoing research on telehealth systems should prioritize user-friendly design, low-tech compatibility, and the inclusion of community health workers.

Our findings are limited by inconsistencies in methodology, quality assessments, and reporting questionnaires, as well as the potential for bias and high heterogeneity in existing reviews [39]. Although many studies were conducted in non-English-speaking countries, only articles published in English were included, which may introduce language bias and result in the omission of relevant non-English-language publications. Future research should aim for larger sample sizes and extended follow-up periods to evaluate the long-term effectiveness of interventions. Details of the studies included in this discussion are summarized in Table 1.

**Table 1:** Summary of reviewed literature

Study	Type	Location	Length	Measurement tools	Participants	Findings
Mena-Iturriaga MJ, et al. (2020) [14]	Secondary Analysis of the 2009-2010 Chilean National Health Survey (NHS)	Chile	October 2009-September 2010	12-Item Short Form (SF-12) questionnaire, Community Oriented Program for the Control of Rheumatic Diseases (COPCORD) Core Questionnaire developed by the International League of Associations for Rheumatology (ILAR)	5276 participants analyzed out of 5293 total surveyees	Chronic musculoskeletal symptoms are prevalent in the Chilean population and affect both physical and mental dimensions of health-related quality of life (QoL), especially in females, older adults, and those with a low level of education.
Williamson J, et al. (2015) [15]	Prospective descriptive study in communities receiving care from a mobile medical clinic	Loreto region of the Peruvian Amazon	Not applicable (N/A)	Spanish-language structured survey tool, qualitative interviews	85 participants	There is a high burden of reported musculoskeletal pain, headaches, and chronic hypertension in Peruvian Amazon communities. Most participants had a limited understanding of their conditions and preferred modern medicine over traditional remedies. Poverty and gender inequality are prevalent, and healthcare access is limited due to long distances and long wait times.
Jacobs R, et al. (2015) [16]	Descriptive, cross-sectional, survey-based study in communities receiving care from brief medical outreach trips	Ben Tre province of Vietnam. Quito and Milpe, Ecuador. Piura, Peru. Santo Tomé, Corrientes Province, Argentina	December 2010-March 2013	Questionnaire offered in Spanish and Vietnamese	847 participants	There is a growing burden of musculoskeletal disorders in developing nations. In both Vietnam and South America, there is a high prevalence of somatic dysfunction, which correlates with poor self-reported health status. These findings may support the integration of osteopathic manipulative treatment (OMT) into conventional medical models. Global treatment protocols should be developed using a population-specific approach, following a needs assessment for musculoskeletal disorders.
Malta DC, et al. (2017) [17]	Cross-sectional study of data from the 2013 Brazilian National Survey of Health (PNS)	Brazil	N/A	Self-reported survey	64,348 households, 60,202 conducted interviews	The prevalence of chronic pain increases gradually and proportionally with age in the Brazilian population. Chronic back pain generates a negative socioeconomic impact by causing work-related disability and hindering daily activities. Specific health interventions should be implemented for population groups with a higher prevalence of back pain.
Queiroz AM, et al. (2022) [18]	Cross-sectional study	Municipality of Manaus, Amazonas, Brazil	N/A	At-home interviews conducted on tablets and smartphones using Research Electronic Data Capture (REDCap)	106 participants	The high prevalence of chronic low back pain (CLBP), limited access to treatment, and varying impacts on daily activities and QoL among elderly individuals residing in rural riverside areas of Brazil should be regarded as a public health problem.
Andrade & Chen (2022) [19]	Cross-sectional analysis of data from the 2019 Brazilian National Survey of Health (PNS)	Brazil	N/A	Self-reported survey	85,687 participants	Chronic back pain is prevalent among Brazilian adults and can be accompanied by severe activity limitations. The prevalence of pain, activity limitations, and treatment are influenced by biological, psychological, and social disparities, which should be addressed in future studies.
Jesus-Moraleida FR, et al. (2017) [20]	Cross-sectional comparison study	Brazil and The Netherlands	N/A	Patient-reported questionnaires, finger-to-floor distance test & “Time Up and Go” test to assess physical function, independent samples t-test, Mann–Whitney U test, Chi-squared test, Statistical Package for Social Sciences (SPSS) software version 22.0 (International Business Machines Corporation)	602 Brazilian and 675 Dutch participants aged ≥55 years	Back pain is disabling in older individuals, and the country of residence is an important determinant of disability and pain in these populations. More severe symptoms are observed in women with poor sleep quality, comorbidities, lower education, and physical inactivity, regardless of country.
Roy AD, et al. (2023) [21]	Editorial article	N/A	N/A	N/A	N/A	The tribal health system in India faces unique challenges. The successful delivery of healthcare services to these populations is hindered by geographical remoteness, inadequate infrastructure, language and cultural barriers, a shortage of healthcare professionals, and socioeconomic disparities. Overcoming these challenges requires collaborative efforts between the government, medical specialists, and indigenous tribes.
Bitter C, et al. (2021) [22]	Retrospective analysis of data from patients who attended a health camp in October, 2017	Thonche Village in the Manang District of Nepal	2 days	Paper clinic intake forms	443 participants	Dental problems were the most common complaint among rural Nepali patients. Analgesics and antibiotics were the most commonly prescribed medications. Understanding the acute care needs of this population is essential for the future utilization of limited health resources.
Maroju RG, et al. (2023) [23]	Narrative review	N/A	N/A	N/A	N/A	India has been integrating technology into healthcare for over a decade in an effort to improve access. Broadband expansion and telemedicine are promising options to enhance access, especially in remote areas of the country. Overall, these efforts aim to address healthcare gaps, improve public health, and provide cost-effective solutions.
Sornoza KE, et al. (2023) [24]	Epidemiological, observational, cross-sectional study	Hospital de los Valles in Quito, Ecuador	January 2017-December 2020	Data from medical records and anonymized patients, Philips Achieva 1.5T magnetic resonance imaging (MRI) to classify Modic changes	288 Ecuadorian patients with Degenerative Disc Disease and modic changes, between the ages of 18 and 99	Common characteristics among patients with Degenerative Disc Disease and Modic changes include an overweight habitus, low physical activity, degenerative and protruding hernias, and injury levels at lumbar vertebra 5 (L5) through sacral vertebra 1 (S1), primarily on the lateral and left sides. Analgesics and neuromodulators were the most frequently utilized post-surgical treatments. Microdiscectomy, discectomy combined with posterior decompression, and both anterior and posterior arthrodesis were the most common surgical procedures. Pain persistence and infection were the most frequent complications. Young men and older women are more predisposed to requiring surgery for lumbar disc herniation. Surgery at an older age carries a higher risk of complications, especially infection.
Baral K, et al. (2016) [25]	Descriptive, qualitative study	Patan Academy of Health Sciences (PAHS) in the Kathmandu Valley of Nepal	N/A	Structured assessments, field reports and evaluations, formative and summative evaluations	Medical students, local community members, health workers, rural leaders and consumer groups	The Community-Based Learning and Education (CBLE) program at PAHS effectively integrates medical education with rural healthcare settings, improving students’ understanding of rural health challenges and fostering positive health-seeking behaviors in communities. The program's success is attributed to strong community involvement in curriculum development, student selection, and evaluation. PAHS plans to expand this approach to other health education programs.
Young L, et al. (2019) [26]	Qualitative study	11 towns in Queensland, Australia	November 2017-February 2018	Semi-structured interviews, NVivo 11 Plus (QSR International Proprietary Limited)	39 participants (14 registrars, 12 supervisors, 13 practice managers)	This study provides evidence to support the development of higher-quality general practice training and supervision in remote areas, where there is greater need for more primary care providers.
Espinoza MA, et al. (2022) [27]	Cost-analysis	Chile	1 year	National Health Survey, Bayesian probabilistic sensitivity analysis, Montecarlo simulation, Markov Model	N/A	The magnitude of the cost in Chile’s approach to chronic pain is associated with increased productivity losses and sick leave payments.
Martí-Salvador M, et al. (2018) [28]	Randomized trial	Spain	12 weeks	Short-Form McGill Pain Questionnaire (SF-MPQ), Visual Analog Scale (VAS), Roland-Morris Disability Questionnaire (RMDQ), Oswestry Disability Index (ODI)	66 participants aged 18-60 years	A statistically significant reduction was observed in the experimental group compared to the sham group for all variables assessed at both week four and week 12. An OMT protocol that includes diaphragm techniques produces significant and clinically relevant improvements in pain and disability in patients with non-specific CLBP, compared to the same OMT protocol using sham diaphragm techniques.
Licciardone JC, et al. (2013) [29]	Randomized, double-blind, sham-controlled, 2 x 2 factorial design clinical trial	Dallas-Fort Worth, Texas	August 2006-September 2010, 8-week period of treatments	100 mm VAS, RMDQ, General Health section of the 36-Item Short Form Health Survey questionnaire (SF-36 GH), number of lost work days in the past four weeks, five-point Likert scale, Mann–Whitney U test, Rothman’s T statistic, SPSS software version 17.0.3 (SPSS Incorporated), Epi Info software version 6.04d (Centers for Disease Control and Prevention)	455 total participants (230 OMT, 225 sham OMT, 233 ultrasound therapy, 222 sham ultrasound therapy)	OMT significantly improves CLBP, with patients in the OMT group more likely to experience moderate to substantial pain reductions compared to those receiving sham OMT. Ultrasound therapy (UST) was not efficacious. Patients in the OMT group reported higher satisfaction and used fewer prescription pain medications.
Rotter G, et al. (2021) [30]	Observational trial	Germany	52 weeks	VAS, SF-12 Physical Component Scale (SF-12 PCS), SF-12 Mental Component Scale (SF-12 MCS), Neck Disability Index Score (NDI), Western Ontario and McMaster Universities Osteoarthritis Index (WOMAC), Disabilities of the Arm Shoulder and Hand (DASH) questionnaire, Lequesne Index to assess the severity of osteoarthritis	40 participants	The study observed beneficial changes during and after OMT, in addition to routine care, in patients with four different chronic musculoskeletal pain conditions.
Lizis P, et al. (2023) [31]	Randomized controlled trial	Poland	5 weeks	Numerical Pain Rating Scale (NPRS), ODI	68 participants aged 30-60 years	Both OMT and Kaltenborn-Evjenth Orthopedic Manual Therapy (KEOMT) decreased low back pain (LBP) and disability; however, the reduction was statistically greater in the OMT group compared to the KEOMT group.
Licciardone JC, et al. (2023) [32]	Respective cohort study	United States	April 2016 - April 2022	National Institutes of Health Minimum Dataset, Patient-Reported Outcomes Measurement Information System, RMDQ	1358 participants	OMT, integrated within medical care provided by osteopathic physicians for CLBP, was associated with improved pain and related outcomes.
Licciardone JC & Aryal S (2014) [33]	Analysis of a randomized double-blind, sham-controlled study	United States	2006-2011, 12-week period of treatments	Kaplan–Meier analysis, p-value, Cochrane Back Review Group criteria, SPSS software version 21 (International Business Machines Corporation), VAS, RMDQ, SF-36, Mann–Whitney U test	455 participants	Among patients with an initial clinical response prior to week 12, those in the OMT group relapsed less frequently than those in the sham OMT group. Overall, 49 patients in the OMT group attained or maintained a clinical response at week 12, compared to patients in the sham OMT group.
Wilson F, et al. [34]	Observational Study	N/A	N/A	2002–2012 Medical Expenditure Panel Surveys	Total 16,546 respondents	Cost-effectiveness analysis suggests that osteopathic, family/general, internal medicine doctors and chiropractors and massage therapists were more cost-effective than other specialties in improving physical function to back pain patients
Verhaeghe N, et al. (2018) [35]	Cost-utility analysis	Belgium	1 year	Incremental cost-effectiveness ratio, Quality-Adjusted Life Years (QALYs)	N/A	Osteopathy was found to be a cost-effective strategy for treating back and neck pain compared to usual care.
De Cola MC, et al. (2020) [36]	Descriptive exploratory study	Italy	1 year	Progressive Healthcare Online Expert Based Organization (PHOEBO®) telemedicine system, ad hoc questionnaire, Likert scale, System Usability Scale (SUS), Shapiro–Wilk test, Mann–Whitney U test, Chi-squared test, Fisher's Exact Test	131 participants	Telemedicine can be useful in improving the health and QoL of disadvantaged older people, particularly those affected by severe comorbidities and living far from healthcare services. Patient satisfaction with the service was rated as good by the majority of participants.
Fatoye F, et al. (2019) [37]	Randomized controlled trial	Nigeria	8 weeks	QALYs, ODI, Incremental Cost-Effectiveness Ratio (ICER)	47 participants	Telerehabilitation for individuals with non-chronic LBP is cost-effective.
Siraj S, et al. (2022) [38]	Simulation-based education workshop	Canada	3 hours, 40 minute sessions	Gamified Educational Networking (GEN), Peer-assisted learning (PAL) strategies, online survey	25 participants	Annual workshops are an ideal solution for ensuring the efficient delivery of simulation training to rural and remote healthcare professionals. Feedback from online surveys will be incorporated into future educational workshops.
Bagagiolo D, et al. (2022) [39]	Overview of systematic reviews and meta-analyses	N/A	N/A	Version 2 of a Measurement Tool to Assess Systematic Reviews (AMSTAR-2), Preferred Reporting Items for Systematic Reviews and Meta-Analyses (PRISMA) statement	N/A	A total of nine articles were reviewed. The systematic reviews and meta-analyses currently available provide promising evidence suggesting OMT’s potential effectiveness in managing musculoskeletal disorders.

## Conclusions

This systematic review aimed to consolidate recent research on the use of OMT, telemedicine, and alternative educational models to address healthcare disparities faced by rural and remote populations with CLBP. The body of research supporting OMT highlights its potential to positively impact the physical, mental, and emotional aspects of health, making it a compelling treatment option for chronic musculoskeletal conditions, which are among the most prevalent health issues in rural areas. The studies reviewed suggest that OMT can be an effective therapy for CLBP when applied appropriately and in the right context. However, variations across methodologies, assessment tools, and quality assessments in literature point to the need for greater standardization in OMT research.

Over the past decade, advancements in OMT research are reflected in the increasing number of randomized placebo-controlled and descriptive studies, indicating a growing body of evidence supporting its efficacy. This trend highlights the need for more controlled trials, longer follow-up periods, and further exploration of the long-term benefits of repeated treatments. As OMT continues to evolve as a viable therapeutic approach, particularly for underserved rural populations, integrating telemedicine, incentivized educational programs, and community-centered healthcare models will be crucial for improving access and engagement.

Understanding the profound impact of chronic musculoskeletal pain on rural communities and tailoring treatment to meet their unique needs should remain a priority. With continued research, OMT, in conjunction with telemedicine and rural health education, has the potential to become a cornerstone of holistic care for rural populations, offering a path to better clinical outcomes and an improved quality of life.
